# Temporal and spatial evolution of grey matter atrophy in primary progressive multiple sclerosis^☆^

**DOI:** 10.1016/j.neuroimage.2013.09.059

**Published:** 2014-02-01

**Authors:** Arman Eshaghi, Benedetta Bodini, Gerard R. Ridgway, Daniel García-Lorenzo, Daniel J. Tozer, Mohammad Ali Sahraian, Alan J. Thompson, Olga Ciccarelli

**Affiliations:** a NMR research Unit, Department of Brain Repair and Rehabilitation, UCL Institute of Neurology, Queen Square MS Centre, London, UK; b MS Research Center, Neuroscience Institute, Tehran University of Medical Sciences, Tehran, Iran; c Brain and Spine institute, ICM, Paris, France; d Wellcome Trust Centre for Neuroimaging, UCL Institute of Neurology, London, UK; e National Institute of Health Research (NIHR), UCLH, Biomedical Research Centre (BRC), London, UK; f Department of Neuroinflammation, UCL Institute of Neurology, Queen Square MS Centre, London, UK; g Centre de Recherche de l'Institut du Cerveau et de la Moelle épinière, Université Pierre et Marie Curie, Inserm U975, Paris, France

**Keywords:** Multiple sclerosis, Voxel-based morphometry, Tensor-based morphometry, Brain atrophy, Longitudinal analysis

## Abstract

Grey matter (GM) atrophy occurs early in primary progressive MS (PPMS), but it is unknown whether its progression involves different brain regions at different rates, as is seen in other neurodegenerative diseases. We aimed to investigate the temporal and regional evolution of GM volume loss over 5 years and its relationship with disability progression in early PPMS.

We studied 36 patients with PPMS within five years from onset and 19 age and gender-matched healthy controls with clinical and imaging assessments at study entry and yearly for 3 years and then at 5 years. Patients were scored on the expanded disability status scale (EDSS) and MS Functional Composite (MSFC) at each time-point. An unbiased longitudinal voxel-based morphometry approach, based on high-dimensional spatial alignment within-subject, was applied to the serial imaging data. The rate of local (voxel-wise) volume change per year was compared between groups and its relationship with clinical outcomes was assessed.

Patients deteriorated significantly during the five years follow-up. Patients showed a greater decline of GM volume (*p* < 0.05, FWE-corrected) bilaterally in the cingulate cortex, thalamus, putamen, precentral gyrus, insula and cerebellum when compared to healthy controls over five years, although the rate of volume loss varied across the brain, and was the fastest in the cingulate cortex. Significant (*p* < 0.05, FWE-corrected) volume loss was detected in the left insula, left precuneus, and right cingulate cortex in patients at three years, as compared to baseline, whilst the bilateral putamen and the left superior temporal gyrus showed volume loss at five years. In patients, there was a relationship between a higher rate of volume loss in the bilateral cingulate cortex and greater clinical disability, as measured by the MSFC, at five years (Pearson's r = 0.49, *p* = 0.003).

Longitudinal VBM demonstrated that the progression of GM atrophy in PPMS occurs at different rates in different regions across the brain. The involvement of the cingulate cortex occurs early in the disease course, continues at a steady rate throughout the follow-up period and is associated with patient outcome. These findings provide new insights into the characteristics of GM atrophy across the brain in MS, and have potential consequences for the selection of brain atrophy as an outcome measure in neuroprotective clinical trials.

## Highlights

• Longitudinal VBM and TBM can be used in longitudinal studies in multiple sclerosis.
• GM loss is a dynamic process in primary progressive multiple sclerosis.
• The highest rate of GM reduction is seen in the cingulate gyri.
• GM atrophy may be used as an outcome measure for neuroprotective clinical trials.

## Introduction

It has recently been proposed that multiple sclerosis (MS) is primarily a neurodegenerative rather than an autoimmune disease (Stys et al., 2012). In primary progressive MS (PPMS), the neurodegenerative element is prominent and the secondary inflammatory reaction is less evident than in the relapse-onset forms (Stys et al., 2012). This neurodegenerative component leads to macroscopic brain atrophy, which may be captured in vivo by brain imaging, providing a potential outcome measure for clinical trials (Barkhof et al., 2012). Grey matter (GM) atrophy is greater in secondary-progressive MS than in relapsing–remitting disease (Miller et al., 2012, Roosendaal et al., 2011) and is an independent predictor of physical and cognitive disability in MS (Calabrese et al., 2013). Additionally, in secondary progressive MS, the loss of volume of GM is greater than that seen in the white matter (WM) (Kapoor et al., 2010) and it shows a more robust relationship with disability than visible WM lesions (Miller et al., 2012).

Despite numerous longitudinal studies on GM atrophy in the different subtypes of MS (Battaglini et al., 2009, Bendfeldt et al., 2012, Ceccarelli et al., 2008, Fisher et al., 2008, Khaleeli et al., 2007), the patterns of dynamic changes in GM over time and their relationship with clinical disability in MS are not well-defined. In a cohort of patients with early PPMS, we used cross-sectional voxel-based morphometry and found that GM atrophy is localized at study entry to specific regions, such as the thalamus and the pre- and post-central gyri (Khaleeli et al., 2007); volume loss in the bilateral thalami, cortical and infratentorial regions was detected during one year follow-up with the same methodology (Sepulcre et al., 2006). However, in PPMS it is not known if the progression of GM atrophy over a longer follow-up involves different brain regions at different stages or with different rates, as is seen in other neurodegenerative conditions, such as Alzheimer's disease (Chetelat et al., 2005) and Huntington's disease (Hobbs et al., 2010).

To investigate the temporal and regional behaviour of GM atrophy over time, it is important to employ the latest techniques that can estimate and localise GM volume changes across all the available time-points, rather than calculating the GM volume independently at each follow-up visit. Additionally, it is preferable not to use the baseline scan as the reference scan in the analysis of longitudinal volume changes, as this can bias the results (Reuter and Fischl, 2011, Yushkevich et al., 2010). These new techniques, called tensor-based morphometry (TBM) and longitudinal voxel-based morphometry (VBM), are considered to be more precise than standard VBM methods (i.e., less variable and less prone to outliers) (Anderson et al., 2012), and have been used successfully in other diseases, such as Huntington's disease (Kipps et al., 2005), frontotemporal dementia (Brambati et al., 2007), and Alzheimer's disease (Hua et al., 2008). The application of TBM to MS has been limited to cross-sectional studies (Aubert-Broche et al., 2011).

In this study, we employed a novel longitudinal VBM approach to describe the temporal and regional evolution of GM volume loss in early PPMS during a five-year follow-up. Additionally, we aimed to investigate the relationship between temporal GM volume changes and clinical outcome.

## Materials and methods

### Subjects

Thirty-six patients with PPMS diagnosed according to Thompson's criteria (Thompson et al., 2000), within 5 years of symptom onset, and 19 age and gender-matched healthy volunteers, were investigated (see Table 1 for demographic and disease characteristics). These patients were a subgroup of the original cohort of PPMS patients who underwent at least one follow-up scan (Khaleeli et al., 2007); eight patients were excluded from this original group to match patients' age to that of a group of healthy controls (Khaleeli et al., 2007). Subjects were assessed longitudinally at baseline, and then after 1, 2, 3, and 5 years (see Supplementary Table 1 for number of participating subjects at each time-point). At each time-point, patients were scored on the expanded disability status scale (EDSS) (Kurtzke, 1983), and the multiple sclerosis functional composite (MSFC) (Cutter et al., 1999), that consists of timed 25-foot walk test (TWT), paced auditory serial addition test (PASAT) and the nine hole peg test (NHPT). The study protocol was approved by Joint Medical Ethics Committee of the National Hospital for Neurology and Neurosurgery, London. Written informed consent was obtained from all participants.

**Table 1:** Demographic and disease characteristics at baseline for patients and healthy controls.

	Patients, N = 36	Controls, N = 19	Significance
Mean age, (CI^a^ 95%)	42.8 (39.4, 46.6)	37.6 (33.4, 41.9)	NS^b^
Gender			NS
Female, N (%)	12 (33%)	9 (47%)	
Male, N (%)	24 (67%)	10 (53%)	
Mean disease duration (CI 95%)	3.3 (2.9,3.6)		
Median EDSS^c^ (minimum, maximum)	4 (1.5, 7)		
Mean MSFC^d^ (CI 95%)	− 1.2 (− 0.7, − 1.6)		

a Confidence interval.

b Non-significant.

c Expanded disability status scale.

d Multiple sclerosis functional composite.

### Image acquisition

All scans were acquired on a1.5 T GE Signa scanner (General Electric, Co, Milwaukee, Wisconsin). At each time point, all subjects underwent T1-weighted 3D fast spoiled gradient recalled imaging (3D-FSPGR) [with 124 contiguous 1.5 mm axial partitions, TR 13.3 ms, TE 4.2 ms, matrix 256 × 256, image in-plane resolution 1.17 × 1.17 mm], and spin echo T2-weighted imaging [with 28 contiguous 5 mm thick axial slices, TR 1720 ms, TE 80 ms, matrix 256 × 256, field of view (FOV) 240 × 240 mm]. The scanner underwent a major upgrade during the study. Thirty percent of the images for healthy controls and 41% of the data for patients were acquired after the scanner upgrade. We adjusted for scanner upgrade in all statistical analysis presented in this work (see below).

### Image analysis

We performed longitudinal VBM following a modification of procedures previously implemented in the SPM software (Wellcome Trust Center for Neuroimaging, UCL, London, www.fil.ion.ucl.ac.uk/spm) (Chetelat et al., 2005, Kipps et al., 2005).

#### Brief overview of TBM and longitudinal VBM

TBM and longitudinal VBM are techniques that use precise non-linear registration over time to determine brain changes; these methods are more powerful for longitudinal studies of neurodevelopmental and neurodegenerative disorders than classical cross-sectional VBM (Ashburner and Friston, 2000), which uses segmentation of tissue classes at each time-point (Anderson et al., 2012). After brain images from different time-points are matched together, a complete analysis of volumetric differences encoded by the spatial transformations can be performed with TBM. Statistical parametric mapping analysis (SPM) for TBM is most powerful with smooth images, but smoothing of the Jacobian determinants reduces the effects by cancelling tissue contraction with neighbouring ventricular expansion. This can be avoided by separating the expanding and contracting regions (Scahill et al., 2002), but this separation has the disadvantage that a group difference in the variability of atrophy could be converted to a (false positive) group difference in mean atrophy. By multiplying relative volumes by segmented GM maps, volumetric changes related to other regions are discounted, and this product gives interpretable estimates of the GM volume for the different time-points. Furthermore, the GM segmentation can be obtained from the within-subject average of multiple time-points increasing signal-to-noise ratio and ameliorating artefacts (Chetelat et al., 2005). Such within-subject processing needs to avoid asymmetries (e.g., towards the baseline image) that can lead to biases and false positive changes (Fox et al., 2011, Thomas et al., 2009, Thompson et al., 2011, Yushkevich et al., 2010). Therefore we adopted the symmetric approach of registering all time-points to unbiased within-subject average images (Reuter et al., 2012, Rohrer et al., 2013).

**Fig. 1:**
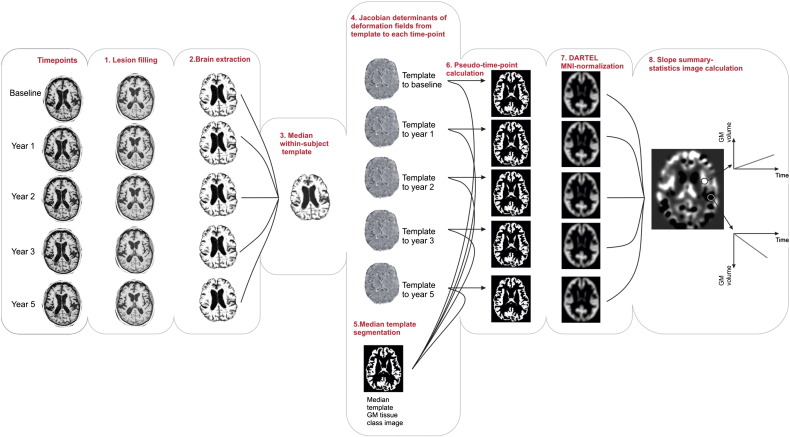
Diagram of the main longitudinal VBM steps performed in first level analysis (see text for more details). VBM; voxel-based morphometry, FSL; FMRIB's Software Library, BET; brain extraction. DARTEL; Diffeomorphic Anatomical Registration using Exponentiated Lie algebra, GM; grey matter.

SPM8, Freesurfer 5.1 (http://surfer.nmr.mgh.harvard.edu/) and FSL 4.1.9 (FMRIB's software library, www.fmrib.ox.ac.uk/fsl) were used for image analysis. The Longitudinal VBM analysis performed in this study consists of the following main steps (Fig. 1):

1. Lesion mask creation and lesion filling:
  T2 lesions were contoured in Dispimage (Department of Medical Physics and Bioengineering, UCL, London) and a binary lesion mask was obtained. T2-weighted images, along with lesion masks, were co-registered to T1-weighted images using rigid registration (SPM8) by maximizing the mutual information of the joint intensity histogram of the images. Lesion masks were used for automatic lesion filling with intensities similar to the normal WM voxels in T1-weighted images, to avoid lesion-associated segmentation errors (Sdika and Pelletier, 2009).
2. Brain extraction:
  All T1-weighted images were corrected for non-uniformity (Sled et al., 1998) and non-brain tissue was removed using FSL BET software with default BET parameters (Smith, 2002). These images were then, if required, manually edited.
3. Unbiased within-subject template construction:
  To avoid asymmetric treatment of time-points, an unbiased within-subject average template was constructed from all available time-points (Reuter and Fischl, 2011, Reuter et al., 2012). The within-subject average template is a voxel-wise intensity median image that is more accurate and insensitive to outliers compared to arithmetic mean templates (Reuter et al., 2010). For template construction, as the first step, a median image was calculated from all time-points. Using a robust and inverse-consistent registration algorithm, all available time-points were registered and re-sampled to the median image (Reuter et al., 2010). A new median image was computed iteratively, until transformations obtained from the previous step converged. This final template was used for further processing. This will be referred to as ‘within-subject template’ throughout this study.
4. Deformation fields and Jacobian determinants:
  Following rigid registration of all time-points to their within-subject template, non-rigid registration was performed using the high-dimensional warping (HDW) (Ashburner et al., 2000) toolbox in SPM8 (regularisation parameter of four with 8 iterations). Deformation fields that map each time-point to the within-subject template were obtained, from which Jacobian (tensor) determinant maps were calculated, showing the contraction or expansion of each voxel relative to the within-subject template image.
5. Segmentation of within-subject templates:
  Within-subject templates were segmented into WM, GM, and CSF tissue maps using the ‘New Segmentation’ toolbox in SPM8. GM tissue class images were used for the rest of our analysis (Weiskopf et al., 2011).
6. “Pseudo-time-point segmentations”:
  In order to restrict the analysis to GM tissue, GM images from within-subject template were multiplied voxel-by-voxel with the HDW Jacobian determinant images that show the amount of volume change. Therefore this step produces images that show the probabilistic volume of GM at each time-point. These product images will be called ‘pseudo-time-points’ henceforth.
7. Spatial normalisation:
  In the next step, GM tissue class images from within-subject templates were fed into DARTEL to create a study-specific group-wise average template (Ashburner, 2007). To account for the unbalanced number of subjects in the patient and control groups, the template was constructed from a balanced set containing all images from the control group with only 19 randomly selected images from the patient group. DARTEL flow fields that warp each subject to the study-specific template were calculated, and then used to normalize all pseudo-time-point images to MNI space with Gaussian smoothing (full width at half maximum 8 mm). Since the focus is on the within-subject longitudinal changes, and modulation may induce multiplicative noise related to intersubject variability of brain shape, which is more pronounced in high-resolution normalisation algorithms (Radua et al., 2014), we chose to proceed without modulation (Thomas et al., 2009).

### Statistical analysis

#### Summary statistic approach for longitudinal data

Unbalanced serial data (i.e. that with differing numbers of unequally spaced time-points) preclude the use of standard repeated-measures ANOVA, whilst voxel-wise imaging data complicate the use of efficient mixed-effects models (cf. Schott et al., 2010). We therefore employ the two-level summary-statistic approach (Matthews et al., 1990), which is similar to the “random-effects” approach commonly employed for functional imaging studies (Friston et al., 2006), and is relatively robust to imbalances such as missing time-points (Mumford and Nichols, 2009).

#### Rate of disability and WM lesion load progression in patients

First, changes in EDSS score were converted to EDSS-steps as described elsewhere (Wingerchuk et al., 1997). In order to identify the annual rates of change in clinical scores, the slopes of linear regressions against time for EDSS and MSFC were calculated using least-squares. A similar approach was used to calculate the rate of WM lesion load accrual over time. To test whether patients had significant disability progression, a one-sample *t*-test was utilized to compare the slope of EDSS and MSFC against the null hypothesis of no progression over time.

#### Temporal and spatial evolution of GM volume loss

For each subject, the slope of a first-level least-squares linear regression against time was computed at each voxel, using all available time-points; this slope image shows the (linear) rate of volume change over time in each voxel. The slope summary-statistic images for all subjects were then taken to the second level for between-subject analysis to investigate dynamic GM changes in both the patient and control groups, and the group difference in atrophy rates. An unbiased explicit GM mask from GM probability maps of the smoothed within-subject templates in MNI space was created for second-level analysis, as described elsewhere (Ridgway et al., 2009). A two-sample *t*-test was used to compare the rates of atrophy between patients and controls. All the above statistical models were adjusted for age, gender and total grey matter volume at baseline as nuisance covariates in the second level model. In addition the proportion of images that were acquired after scanner-upgrade was obtained for each subject and modelled as nuisance covariate in the second level.

To test whether there was an acceleration or deceleration in the rate of GM volume change over time, the quadratic term from a second-degree polynomial regression against time was calculated for each subject and submitted to second-level between-subject analysis.

Results were corrected for multiple comparisons using threshold-free cluster enhancement (TFCE) (*p* FWE < 0.05 with 5000 permutations) as implemented in FSL randomise (Smith and Nichols, 2009).

#### Post-hoc analysis of year-by-year volume loss in patients without missing time-points

To describe the changes in volume occurring year by year, we subtracted GM probability at each time-point from that of baseline in 15 patients who attended all the follow-up visits. The difference images were then fed into FSL randomise and one sample *t*-test was performed (5000 permutations). Results were corrected for multiple comparisons across space using voxel-wise FWE (*p* < 0.05) (without TFCE).

#### Relationship between volume changes and clinical findings

To investigate the relationship between GM volume changes and progression of disability, we correlated the slopes of EDSS, MSFC and T2 lesion-load that represent the annual rates of change, with the GM volume slope images. Additionally, to assess the association between the GM volume loss rate and long-term clinical outcome, a correlation analysis was performed between the rate of GM volume loss and EDSS and MSFC at 5 years. Also, a partial correlation analysis between the rate of GM volume decline and MSFC at 5 years, adjusting for normalized GM volume (SIENAX FSL 4.1.9, http://fsl.fmrib.ox.ac.uk/fsl/fslwiki/SIENA), was performed to test whether the association between atrophy rate and disability at the end of the study holds after accounting for baseline differences of pathology.

## Results

### Rate of disability and WM lesion load progression in patients

Patients progressed during the five-year follow-up, with a mean decrease of 0.25 ± 0.39 (mean ± standard deviation) in MSFC per annum (*p <* 0.001), and a mean increase of 0.36 ± 0.71 in EDSS per annum (*p =* 0.01). There was a mean increase of 2.59 ± 3.41 ml per annum in WM lesion load.

### Temporal and spatial evolution of GM volume loss

**Fig. 2:**
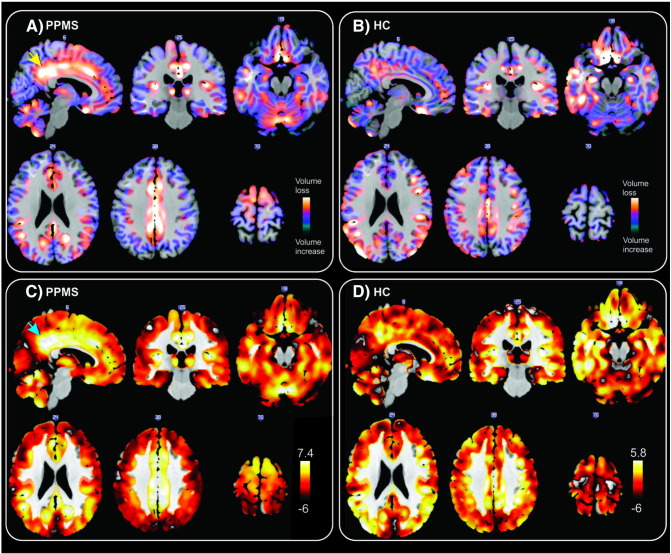
Linear rate of GM volume reduction in patients (A) and in healthy controls (B) adjusted for age, gender, total grey matter volume and the scanner upgrade. Bottom row: Unthresholded one-tailed *t*-statistic map for negative linear effect of time adjusted for nuisance covariates (see above) in patients with PPMS (C) and healthy controls (D). The posterior cingulate gyri and adjacent precuneus (arrows) show the highest rate of volume loss in the patient group. PPMS; primary progressive multiple sclerosis, HC; Healthy controls.

**Fig. 3:**
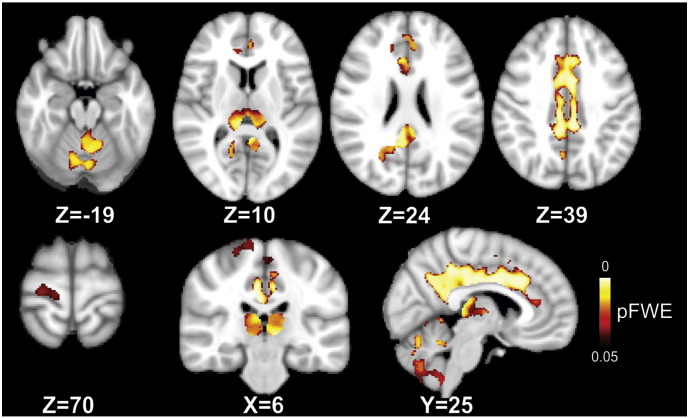
Significant regions of annualized GM loss over 5 years (most informative slices are shown). Two-sample *t*-test shows significantly greater rate of atrophy in the cingulate cortex, precentral gyrus, thalamus, cerebellum, and insula in the patients as compared to controls. Coordinates are in MNI space. Results are adjusted for age, gender and scanner-upgrade and corrected for multiple comparisons across space with *p* FWE < 0.05, threshold-free cluster enhancement. FWE; Family-Wise Error.

The rate of volume loss was not uniform across the brain, but affected different GM structures with variable rates (as shown in Fig. 2); it appeared to be the fastest in the bilateral cingulate gyri and the slowest in the pre-central gyrus. Patients showed a greater rate of reduction in GM volume in the bilateral cingulate gyri and the adjacent precuneus, the cerebellum, the bilateral precentral gyri, the bilateral thalami, and the insula, in comparison with healthy controls over the five-year period (Table 2 and Fig. 3) (*p* FWE < 0.05, TFCE). There was no significant acceleration or deceleration in GM volume change (i.e., change in the rate of atrophy over time) in patients or controls (analogous to one-sample t-tests on the quadratic terms), and no significant group difference between patients and controls (analogous to a two-sample *t*-test on the quadratic terms).

**Table 2:** Regions that show a statistically significant greater rate of GM atrophy in patients than in controls.

Peak location	pFWE	*t*-Value	Cluster size (voxels)	Peak MNI coordinates (*mm*)		
				*X*	*Y*	*Z*
Anterior cingulate gyrus	0.004	4.87	14,919	7.5	16.5	30
Left thalamus	0.009	5.34	2272	− 7.5	− 31.5	1.5
Left putamen	0.031	4.59	351	− 19.5	3	− 3
Right precentral gyrus	0.041	3.61	305	22.5	− 22.5	69
Left precentral gyrus	0.046	3.11	181	− 9	− 19.5	63
Right insula	0.035	5.33	77	31.5	9	− 3

Corrected for multiple comparisons across space with *p* FWE < 0.05, threshold-free cluster enhancement.

### Post-hoc analyses of year-by-year volume loss in patients without missing time-points

In patients, there was no significant volume loss at first and second year when compared to baseline. A significant volume loss in the left insula, left precuneus, and right cingulate cortex was detected at three years, whilst the bilateral putamen and the left superior temporal gyrus showed volume loss subsequently, at five years (see Table 3 for further details).

**Table 3:** Post-hoc analysis of GM volume loss at different stages of early PPMS.

Peak location	pFWE	*t*-Value	Cluster size (voxels)	Peak MNI coordinates (mm)		
				*X*	*Y*	*Z*
*Year 3 vs. baseline*						
Left insula	0.005	5.6	100	− 43.5	− 36	15
Left precuneus	0.004	5.68	72	− 13.5	− 60	12
Right cingulate gyrus	0.018	5.19	49	10.5	− 45	36

*Year 5 vs. baseline*						
Left putamen	0.001	6.47	165	− 19.5	9	− 6
Superior temporal lobe/left insula	0.003	5.83	152	− 51	− 40.5	21
Right putamen	0.004	5.7	102	22.5	15	− 4.5
Right cingulate gyrus	0.003	5.86	100	10.5	− 45	36
Left precuneus	0.019	5.2	22	− 13.5	− 61.5	13.5

Corrected for multiple comparisons across space with *p* FWE < 0.05 at voxel level.

### Relationship between volume changes and clinical findings

**Fig. 4:**
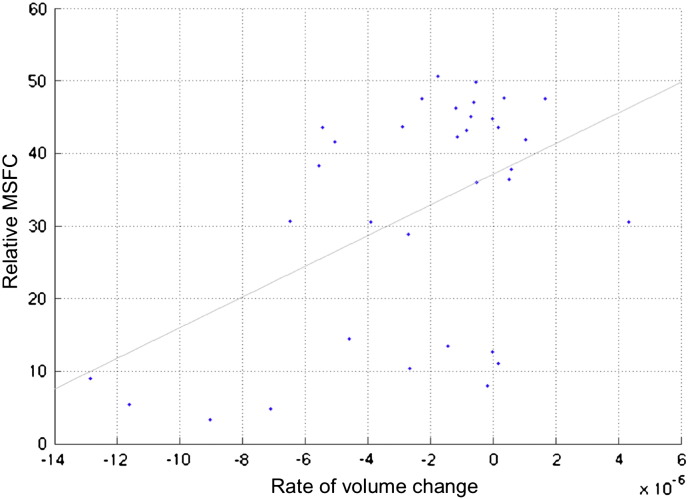
Scatterplot and trend line showing the association between the rate of volume loss in posterior cingulate cortex (AAL atlas) and MSFC at 5 years (r = 0.49, *p* = 0.003). More negative values on *X*-axis represent higher rate of volume loss. MSFC scores are Box–Cox transformed with a shift parameter of 10 to improve normality. Two outliers have been excluded from the analysis (Pearson's r = 0.53, *p* < 0.001 without excluding outliers). The correlation holds after correction for baseline GM normalized volume (Pearson's r = 0.51, *p* = 0001). AAL; Automatic Anatomical Labeling.

In patients, a greater rate of volume loss in the bilateral cingulate gyrus was associated with worse MSFC at 5 years (r = 0.49, *p* = 0.003), (Fig. 4). In addition, this association persisted after correction for baseline normalized GM volume (r = 0.51, *p* = 0.001).

There was no significant association between the rate of GM volume loss and the rate of EDSS step-change, MSFC change, EDSS at 5 years and rate of T2 lesion load accrual per annum.

## Discussion

The four key findings of this study were (i) there was a marked progression of atrophy in several GM regions in patients when compared to healthy controls; (ii) the progression of GM atrophy in patients occurs at different rates in different regions across the brain; the fastest atrophy is seen in the cingulate cortex and the slowest in the precentral gyrus; (iii) some regions showed a significant volume loss at a later time point than other regions; (iv) there was a significant association between rate of volume loss in the cingulate cortex and worse clinical outcome at five years. We will discuss these results in turn.

The findings of including the bilateral cingulate gyri with the adjacent precuneus, cerebellum, bilateral precentral gyri, and bilateral thalami, and insula, are broadly consistent with our cross-sectional and longitudinal studies in the same cohort (Ingle et al., 2005, Khaleeli et al., 2007, Khaleeli et al., 2008, Sastre-Garriga et al., 2005, Sepulcre et al., 2006) and with previous studies from different centres in other types of MS (Audoin et al., 2010, Battaglini et al., 2009, Bendfeldt et al., 2012, Ceccarelli et al., 2008, Fisher et al., 2008). However, we used for the first time an unbiased and novel longitudinal VBM approach, which is more precise than standard methods of analysis of volume changes.

The most interesting finding of our study is that the volume loss observed in the regions mentioned above did not occur at the same speed throughout the brain, but involved different regions with variable rates. The highest speed of GM volume loss was seen in the cingulate gyri with adjacent precuneus. This region showed a significant atrophy in patients at 3 years when compared with baseline, and the rate of volume loss of this region was greater in patients with higher neurological disability at 5 years. Distinctive GM atrophy in the cingulate cortex of early PPMS patients can be interpreted in light of the ‘use-it-and-lose-it’ principle (Geurts and Barkhof, 2008). Posterior medial cortical structures (including the posterior cingulate cortex and the ventral precuneus) are among the most metabolically active regions that are part of the default mode network (DMN) of the brain (Raichle et al., 2001, Zhang and Raichle, 2010). The DMN supports internally directed mental activity and plays an important role in the regulation of cognitive functioning (Nakano et al., 2013). The DMN shows connectivity changes from early stages in MS (Roosendaal et al., 2010), but whether it has a compensatory or maladaptive role has not yet been clarified. A high metabolic rate along with an increased demand for energy to repair axonal and cellular damage following demyelination, and a decreased energy supply secondary to mitochondrial dysfunction with consequent reduction in ATP production from respiratory chains (Lassmann et al., 2012), may induce a chronic state of virtual hypoxia (Trapp and Stys, 2009) with a subsequent axonal vulnerability. Additionally, the cingulate region, especially in its anterior portion, is known to contribute to motor control, via its connections with the spinal cord and motor cortex; therefore, a higher rate of atrophy of this region may be expected to lead to greater physical and cognitive disability, as assessed by the MSFC, at 5 years. However, there was no correlation between the rate of GM volume reduction and the rate of MSFC change; this can be explained by the complex relationship between functional and structural changes in MS (Tomassini et al., 2012) and by the role played by the GM lesions (Calabrese et al., 2013), which were not included in our study. Additionally, Fig. 4 shows that some patients were more disabled than others despite having the same rate of atrophy; this suggests that there may be factors that contribute to the disability in addition to GM atrophy, such as spinal cord damage, which are not investigated in this study, and that clinical scores may have a limited ability to reflect the real extent of neurological disability (Barkhof, 2002).

Our previous cross-sectional VBM analysis in the same patient cohort has shown volume changes in the pre-central cortex and thalamus in patients with PPMS when compared to healthy controls (Khaleeli et al., 2007). We have extended this observation by demonstrating that the progression of atrophy continues in these regions in patients over 5 years. Preferential involvement of the particularly long projections from pyramidal neurons can account for axonal and neuronal loss in the pre-central gyrus (Narayanan et al., 1997), although this region showed the slowest progression of atrophy. This may be explained by B-cell follicle-like structures (which are harbinger of meningeal inflammation and subsequent tissue loss) that could be frequently found in the deep sulci of the forebrain (cingulate, temporal and insular areas) in people suffering from SPMS (Howell et al., 2011). Also, we found a concurrence between regions with higher metabolism and higher rate of atrophy and postulated that this might be attributed to energy failure and mitochondrial dysfunction in MS (see above). Most of these could be found in regions affected by progressive atrophy — areas other than the precentral gyrus (e.g. cingulate cortex, insula, thalamus). A wealth of evidence implicates particular vulnerability of deep GM structures (mainly the thalamus) in MS (Lassmann et al., 2012, Sepulcre et al., 2006). Thalami act as the hub of important brain networks (highly interconnected with a plethora of cortical regions), subserving a spectrum of functions ranging from motor to cognition and consciousness (van den Heuvel and Sporns, 2011). This inevitably renders the thalamus vulnerable to diffuse MS-associated pathological processes (Minagar et al., 2013).

A significant progression of atrophy over 5 years was seen in patients in the cerebellum. The cerebellum is a major predilection site for demyelination especially in progressive subtypes of MS that usually escapes detection by conventional MRI studies (Kutzelnigg et al., 2007). Indeed, the deep meningeal infoldings in the cerebellum could accommodate inflammatory processes for a prolonged period of time, which could lead to the final occurrence of atrophy in the cerebellar cortex. Our post-hoc model performed on a subset of 15 patients with no missing data did not show GM loss in the cerebellum, but this might simply reflect reduced statistical power in comparison with the two-level summary statistics approach that included all available time-points for all subjects.

An interesting observation of our study is that significant atrophy in the putamen bilaterally and in the left superior temporal gyrus is observed at year 5 when compared to baseline, which is at a later time point compared with the volume loss occurring in other regions, such as the cingulate cortex, the insula and the precuneus. Subpial demyelination affects areas with restricted cerebrospinal fluid circulation that can explain earlier GM volume reduction in the cingulate and insular cortices that we found in this cohort, suggesting an effect from stagnant mediators of adjacent meningeal inflammation (Kutzelnigg et al., 2007). Moreover, the cingulate cortex, insula and the temporal lobe have been shown to have the most frequent cortical lesions (Bo et al., 2003, Vercellino et al., 2005). Insular atrophy was more pronounced in its posterior section, which is more densely myelinated and have prominent connections to the premotor cortex, superior parietal lobule (precuneus) and cingulate regions (Flynn, 1999). These results show that how future connectomic research may play a role to further our understanding of the pathological substrates of neurodegeneration in MS (Griffa et al., 2013).

When looking at the temporal and spatial progression of atrophy in other neurodegenerative and neuropsychiatric disorders a similar picture of accelerated GM loss may emerge: the cingulate cortex and temporal lobe in persons with Alzheimer's and Parkinson's disease (Chetelat et al., 2005, Ramirez-Ruiz et al., 2005), subcortical structures such as thalamus in individuals with Huntington's disease and frontotemporal dementia (Hobbs et al., 2010, Mahoney et al., 2012), and superior temporal lobe in persons with schizophrenia (Vita et al., 2012). Higher involvement of certain regions over time strengthens the hypothesis of a specific relevance of these regions in the pathophysiology of each disorder. Overall, our results may suggest that similar to other neurodegenerative and neuropsychiatric disorders, atrophy is more pronounced in specific regions of the brain in people with PPMS and may progress chronologically so that the cingulate cortex and the precuneus are affected earlier than the putamen, although the number of patients attending all five time points was small.

A methodological consideration is that we used registration-based pseudo-time-points, which tend to underestimate biological changes, and are more computationally expensive compared to the separate segmentation and subtraction of the time-points. However, our method provides a lower variance for estimation of the atrophy rate, is less susceptible to segmentation errors, and is more statistically powerful than separate segmentation of time-points (Anderson et al., 2012). Additionally, we utilized a symmetric approach of registering to a median intensity image as a template to reduce bias related to asymmetry, as well as the effects of outliers (e.g., due to movement), which is an important improvement in comparison with previous works. Future research could further improve longitudinal VBM and TBM methods (Ashburner and Ridgway, 2012). Another limitation common to all voxel-based methods (including SPM, VBM and TBM) is that differences between regions are not formally tested for significance (Jernigan et al., 2003). This means that apparent regional differences should be interpreted cautiously, particularly with regard to non-significant regions, since sensitivity varies across the brain due to variation in the accuracy of registration and segmentation (Pereira et al., 2010, Ridgway et al., 2008). It should also be noted that summary-statistics approach to mixed-effects modelling is most effective for highly structured data (i.e., similar observations for all subjects). Moreover, real mixed-effect models might improve the sensitivity of the second level inference by accounting for the variable precision of within-subject measurements. Finally, since non-linear registration algorithms are inherently less stable than linear registration algorithms a possible adjunct for future studies is to impose a penalty term in high dimensional warping related to the proximity and quantity of GM lesions seen on more sensitive MR sequences (e.g. DIR).

In conclusion, using a novel longitudinal VBM approach we demonstrated that the rate of GM atrophy in early PPMS is not identical throughout the brain. This is relevant for the development of imaging outcome measures in neuroprotective clinical trials, which commonly employ total GM and whole brain atrophy. Regions characterised by a particularly active metabolism, higher interconnection with other brain regions, and possibly affected by overlying meningeal inflammation, such as the cingulate cortex, may be particularly vulnerable to atrophy and their volume loss may be associated with clinical outcome.

The following are the supplementary data related to this article.

Supplementary Table 1 Individuals assessed at each time-point.
