# Supplementary material for: Temporal and spatial evolution of grey matter atrophy in primary progressive multiple sclerosis
Source: Neuroimage. 2014 Feb 1;86:257–64. doi: 10.1016/j.neuroimage.2013.09.059 (PMC3898881; doi:10.1016/j.neuroimage.2013.09.059)
Supplement: Supplementary Table 1 — Individuals assessed at each time-point. [file mmc1.docx]

**Supplementary Table 1.** Individuals assessed at each time-point

| Group | Baseline | Year 1 | Year 2 | Year 3 | Year 5 | Subjects with no missing time-point |
| --- | --- | --- | --- | --- | --- | --- |
| Patients | 36 | 30 | 27 | 27 | 25 | 15 |
| Controls | 19 | 15 | 14 | 10 | 15 | 5 |
